# Supplementary material for: Prevalence and detection of low-allele-fraction variants in clinical cancer samples
Source: Nat Commun. 2017 Nov 9;8:1377. doi: 10.1038/s41467-017-01470-y (PMC5680209; doi:10.1038/s41467-017-01470-y)
Supplement: Supplementary file 1 — Supplementary Information [file 41467_2017_1470_MOESM1_ESM.pdf]

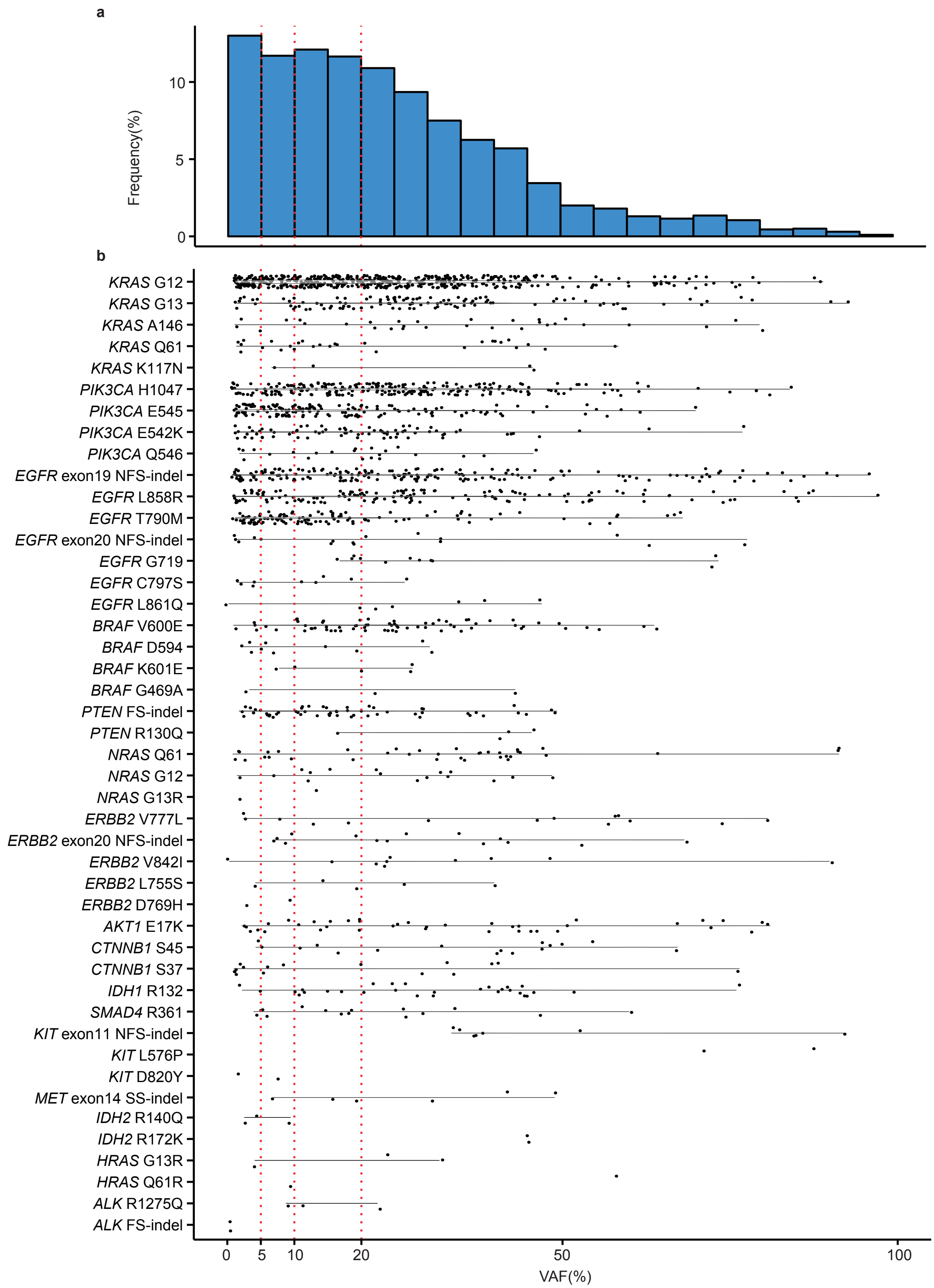

Supplementary Figure 1. VAF distributions for actionable SNVs and indels in our cohort. (a) A histogram of VAFs for actionable variants. **(b)** VAF distributions of individual actionable variants. Only the variants observed more than once are displayed here. NFS, non-frameshift; SS, splicing site; FS, frameshift.

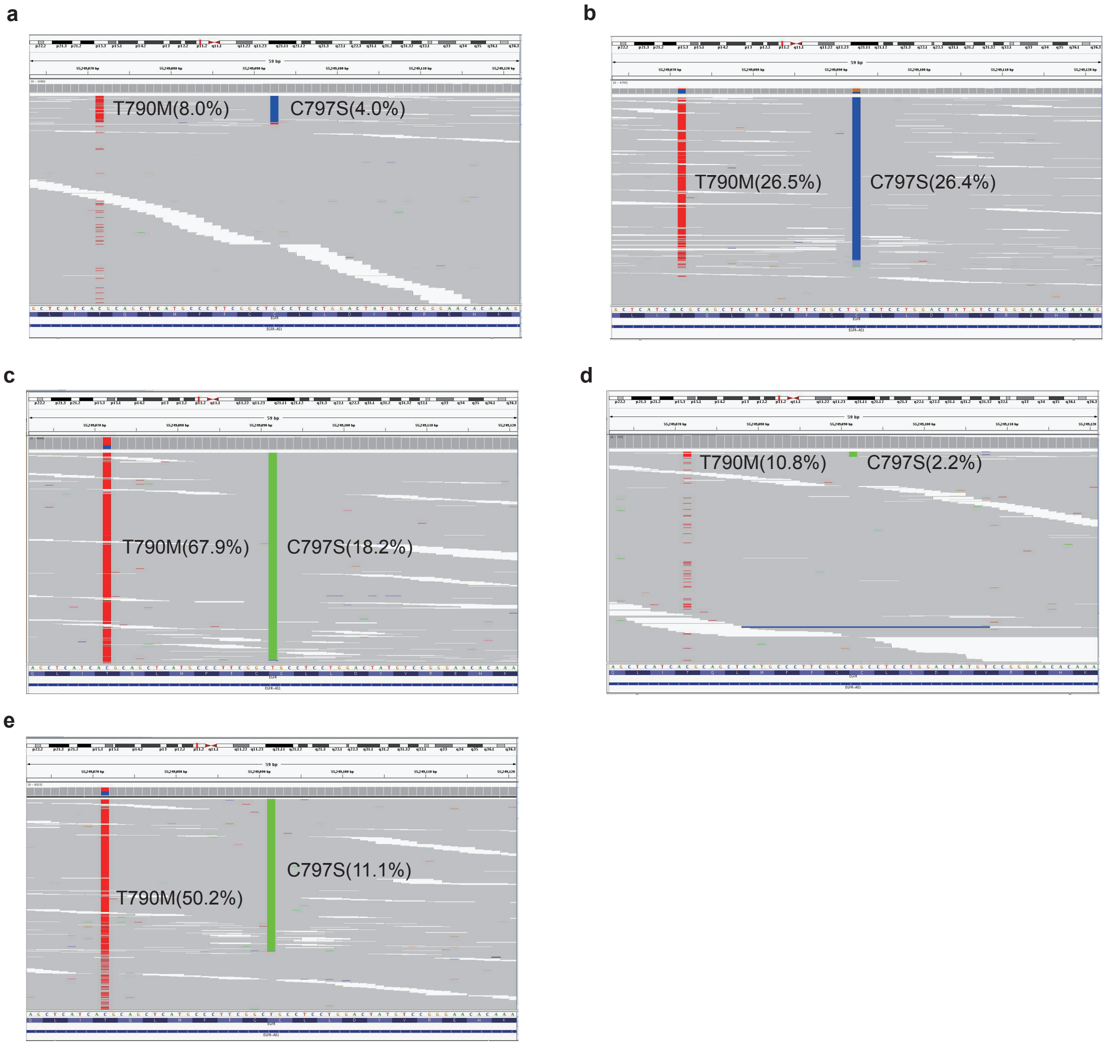

Supplementary Figure 2. *EGFR* C797S mutations in refractory lung cancer samples.
All *EGFR* C797S mutations occurred in *cis* with *EGFR* T790M mutation and at lower VAFs.

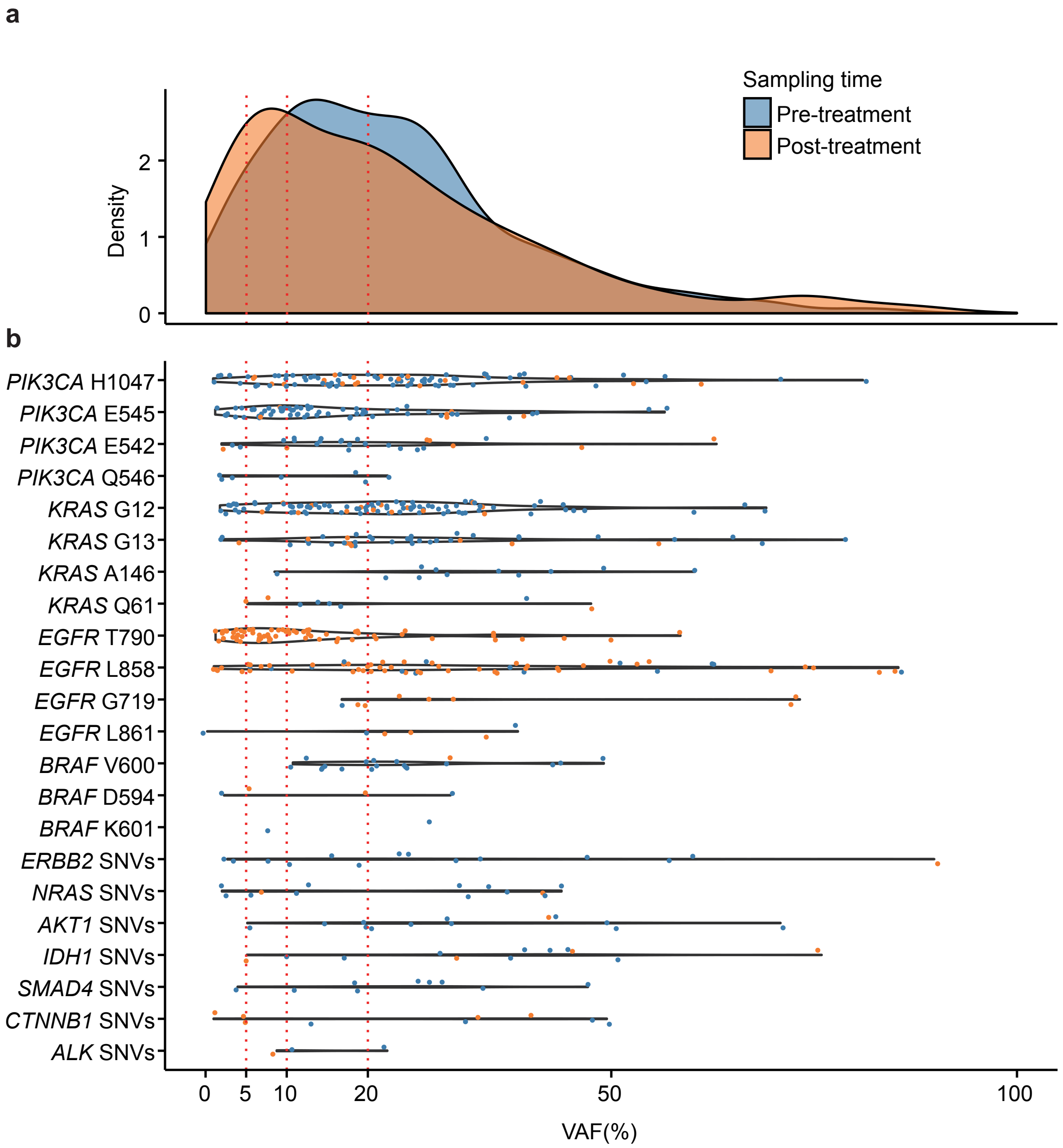

Supplementary FIgure 3. Comparison of VAF distributions for actionable SNVs in samples classified as pre- and post-treatment (chemotherapy). (a) VAF distributions for the two groups are shown after kernel density smoothing. There is no significant difference between the two distributions ($p > 0.05$, wilcoxon rank sum test). **(b)** VAF distributions of individual actionable variants, colored by their sampling time. The mutations that were observed more than once are summarized here.

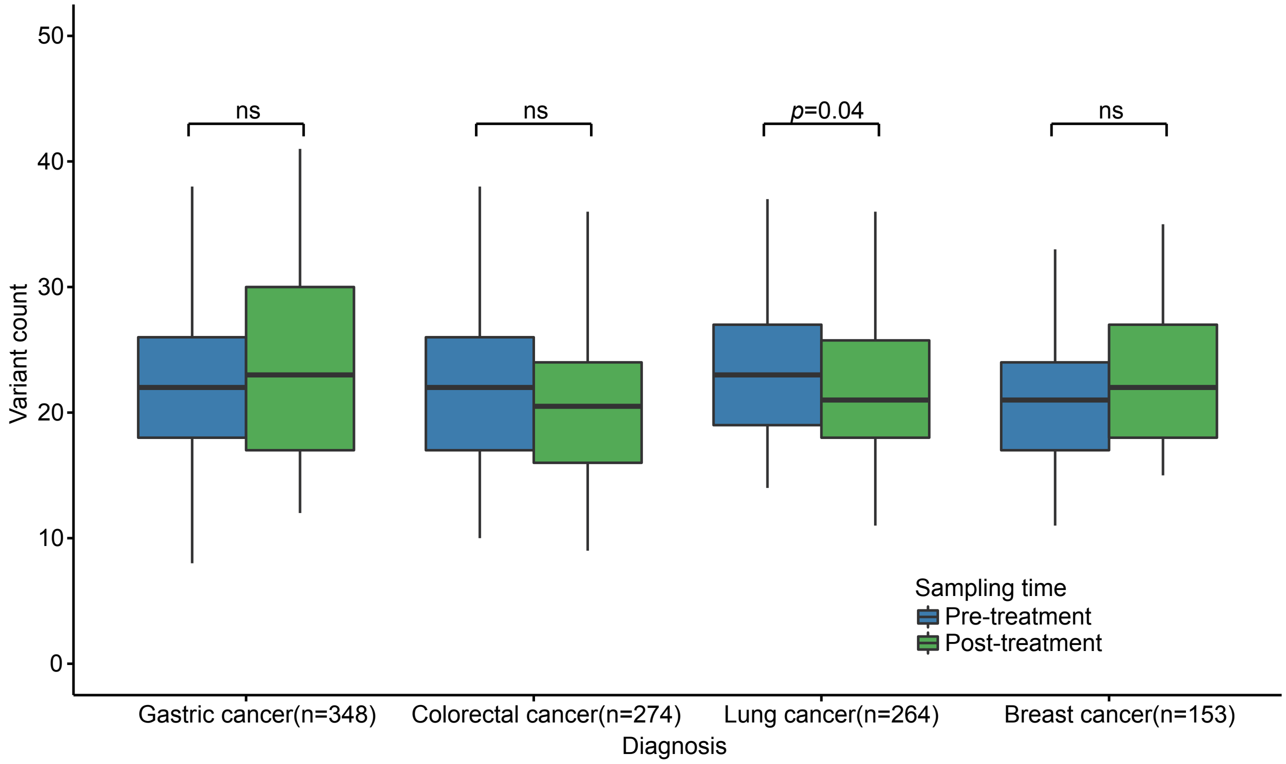

Supplementary Figure 4. Comparison of pre- and post-treatment variant counts in four cancer types. Only those samples profiled on the V2 platform were used; the *p* values were calculated using the Wilcoxon rank sum test. ns, not significant.

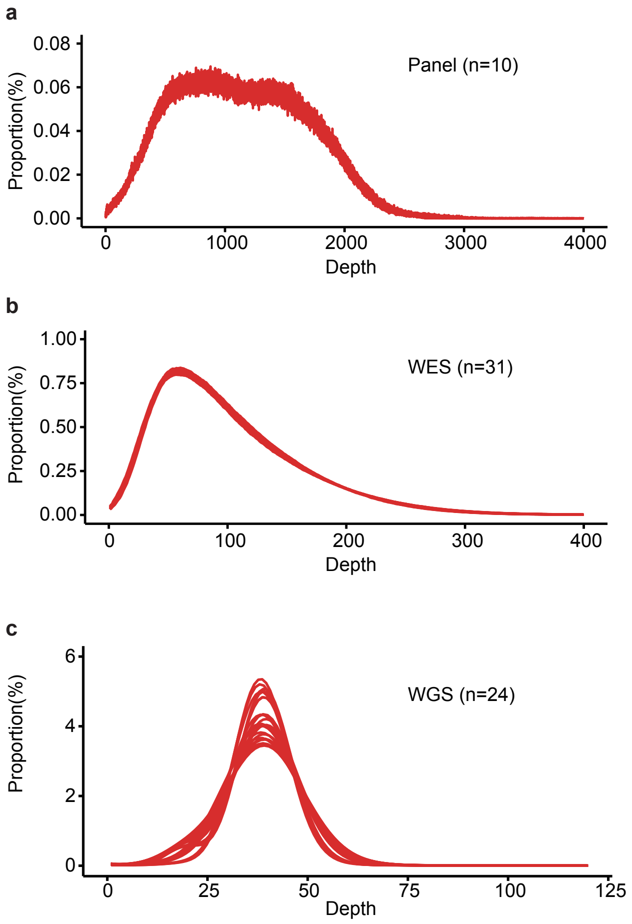

Supplementary Figure 5. Comparison of genome coverage distributions for panel, whole-exome sequencing (WES), and whole-genome sequencing (WGS) data in normal samples. (a) 10 normal cell lines, sequenced by the 381-gene CancerSCAN panel. **(b)** 31 normal blood samples, sequenced by WES (SureSelect XT Human All Exon v5 kit). **(c)** 24 normal blood samples, sequenced by WGS (TruSeq Nano kit). All sequencing was performed on Illumina HiSeq 2500. Coverage of each sample was normalized by the median depth of the samples processed on each platform. Samples used are listed in **Supplementary Data 2** and **Supplementary Table 1**.

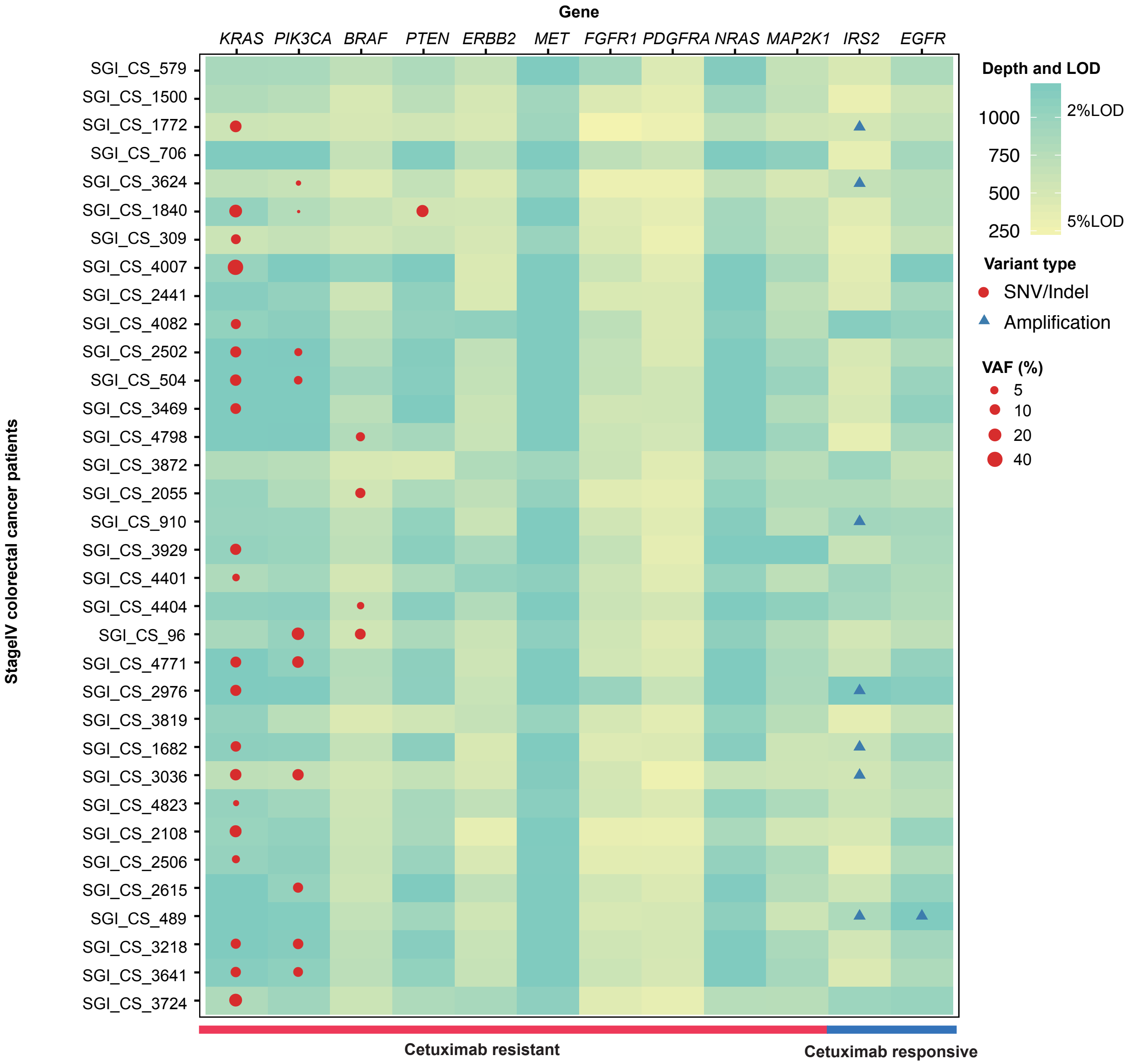

Supplementary Figure 6. Limit of detection estimates for genomic alterations. Providing the limit of detection (LOD) estimates based on observed sequencing depths can be informative. For example, anti-EGFR therapy (Cetuximab) is given to Stage IV colorectal cancer patients only if they are wild-type for RAS. The heatmap shows the mutations in genes associated with response to anti-EGFR therapy[1], with the size of the circles corresponding to the VAFs and LODs indicated by the background color. By annotating the detection results with LODs, it becomes easier to distinguish between true negatives and false negatives.

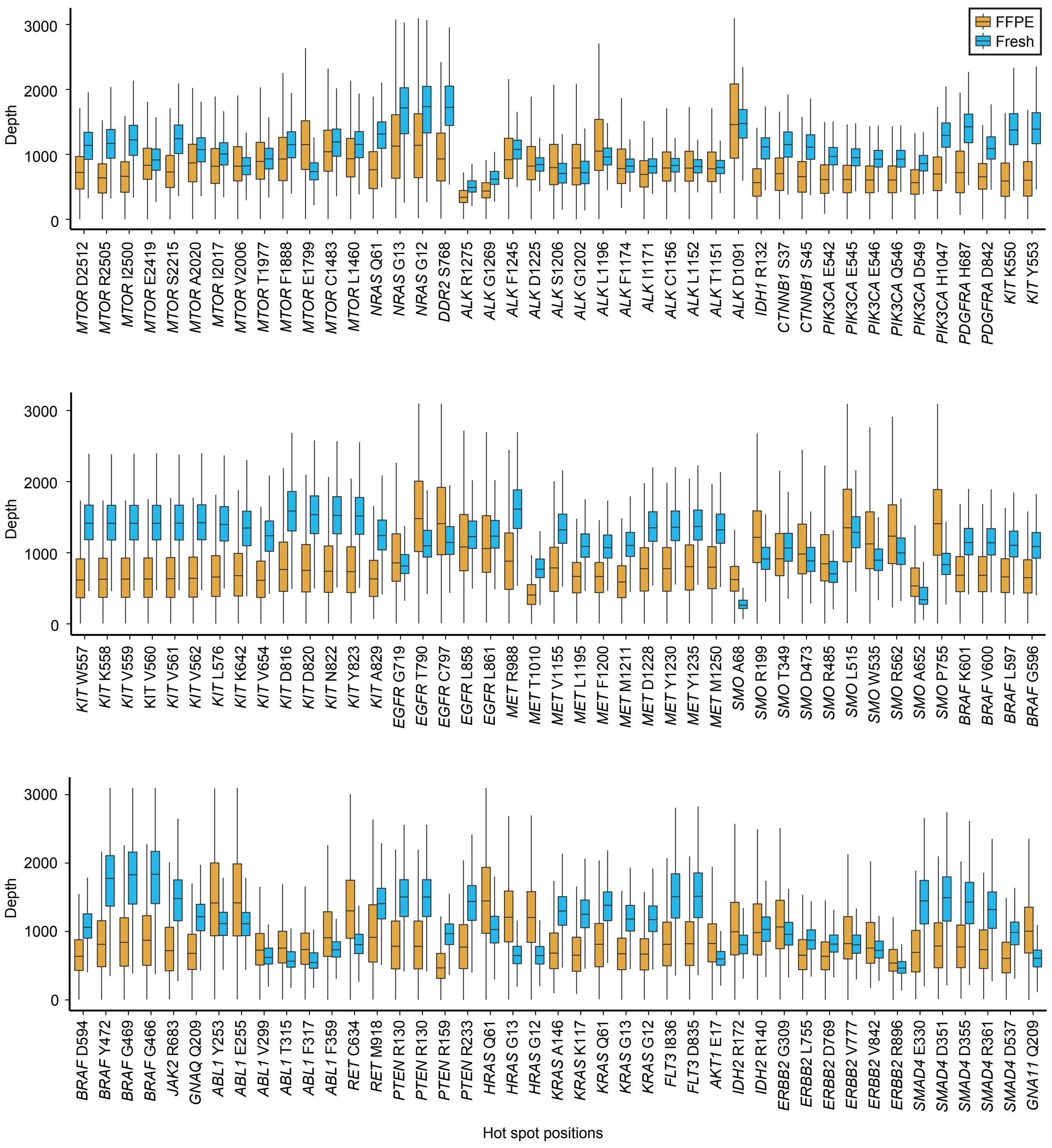

Supplementary Figure 7. Difference in sequencing coverage between FFPE and fresh samples. Distribution of read depths at 241 SNV hotspot positions (Tier 1) are shown for 5095 samples (2512 FFPE samples and 2583 fresh samples). The average coverage is generally higher for fresh samples but there is substantial variability across genes.

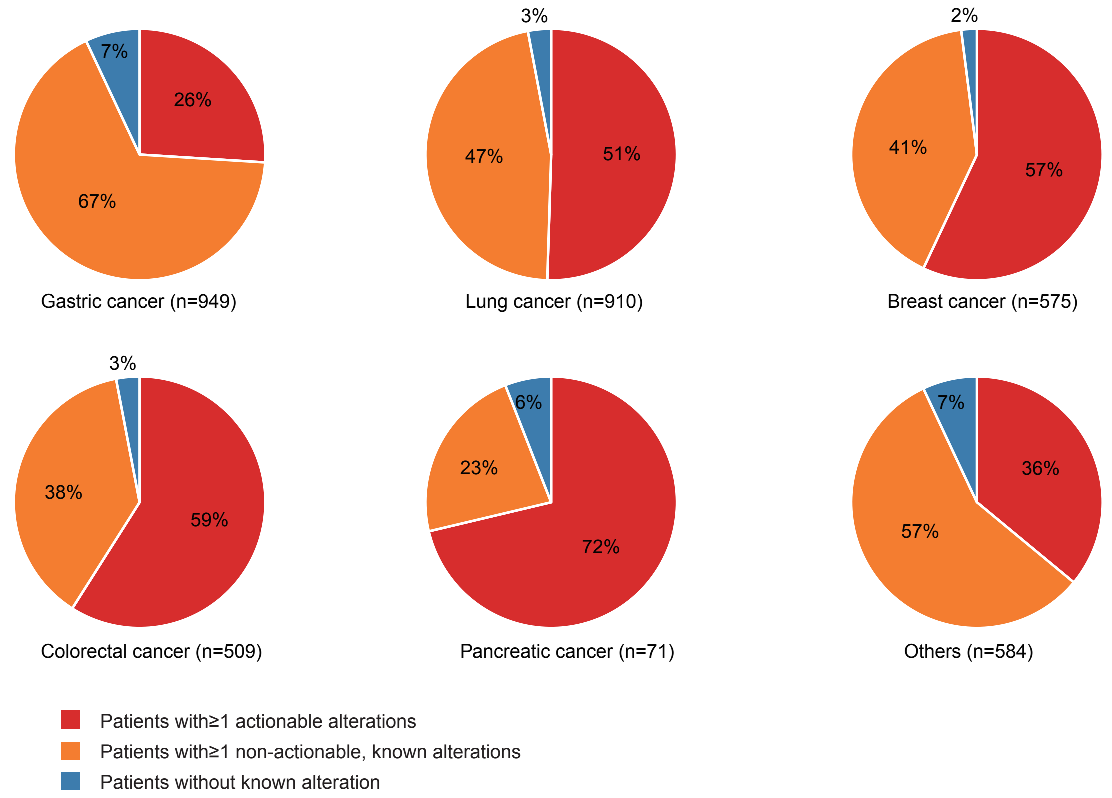

Supplementary Figure 8. Proportion of patients (panel V2, n=3598) with actionable, known but non-actionable, and without known alterations. The overall distribution is shown in **Fig. 4c**. Here, the proportions are shown separately for the five most common tumor types and others. “Known” alterations are those found in COSMIC.

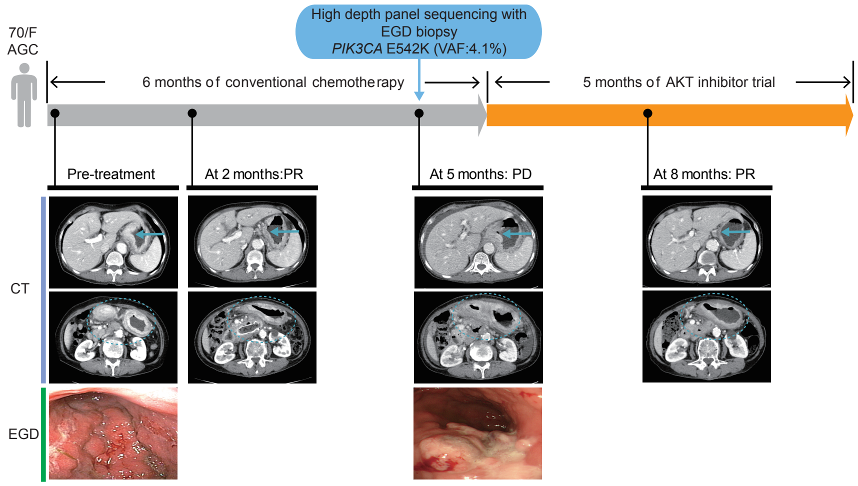

Supplementary Figure 9. Second example of a patient with a clinically-relevant low-allele-fraction mutation. A 70-year old female patient (SGI_CS_2769) had a metastatic gastric cancer with peritoneal seeding. After failing to respond after 8 cycles of capecitabine/oxaliplatin chemotherapy, she had an esophagogastroduodenoscopy (EGD) biopsy. Genomic profiling of the biopsy tissue revealed a *PIK3CA* E542K mutation with 4.1% VAF. The variant was validated by dPCR (**Supplementary Data 4**). The patient was enrolled onto an AKT inhibitor trial, and has achieved partial remission for 5 months. Arrows on the CT (computed tomography) images indicate the location of the tumor and the dotted circles indicate regions of peritoneal seeding. AGC, advanced gastric cancer; PD, progressive disease; PR, partial response.

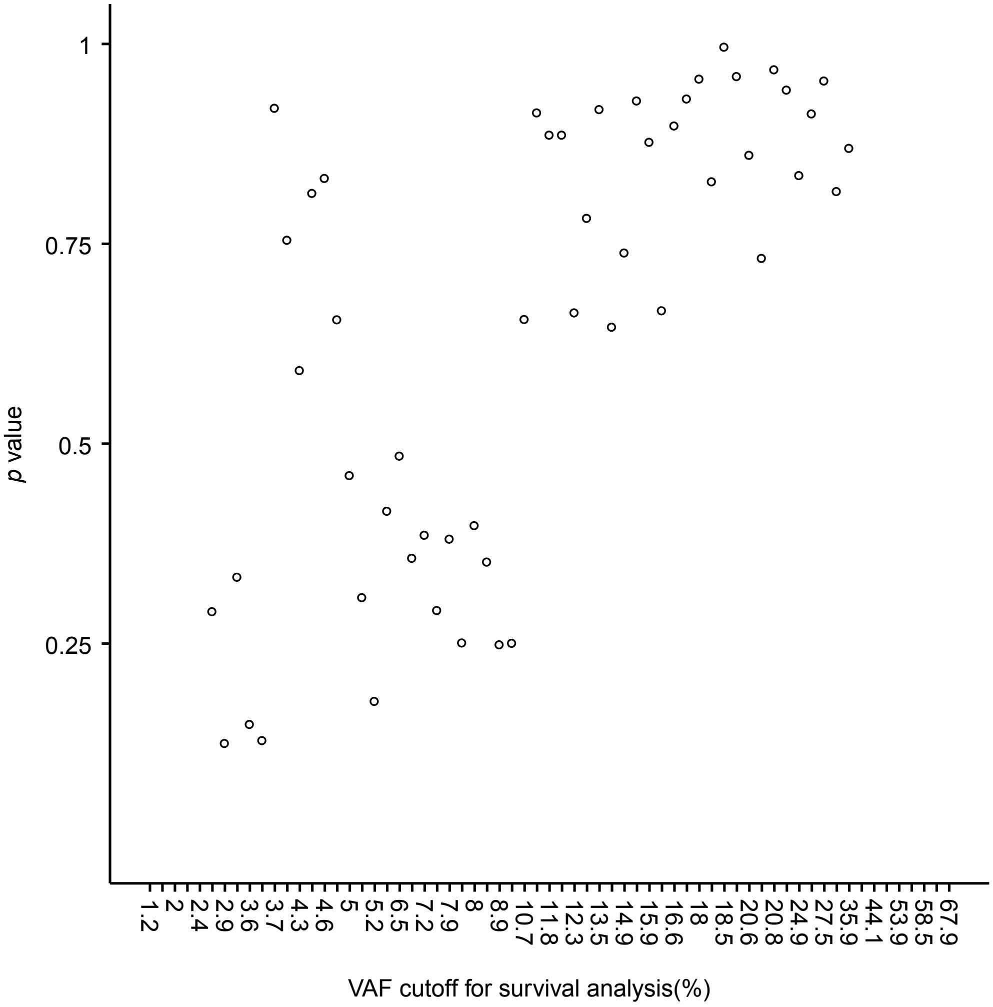

Supplementary Figure 10. Robustness to the VAF cut-off for survival analysis. In **Fig. 5e**, 5% VAF was used as the cut-off value for comparing the survival curves of low *vs* high-VAF cases. This plots shows that the *p* values of log-rank test are non-significant for all possible divisions into two groups.

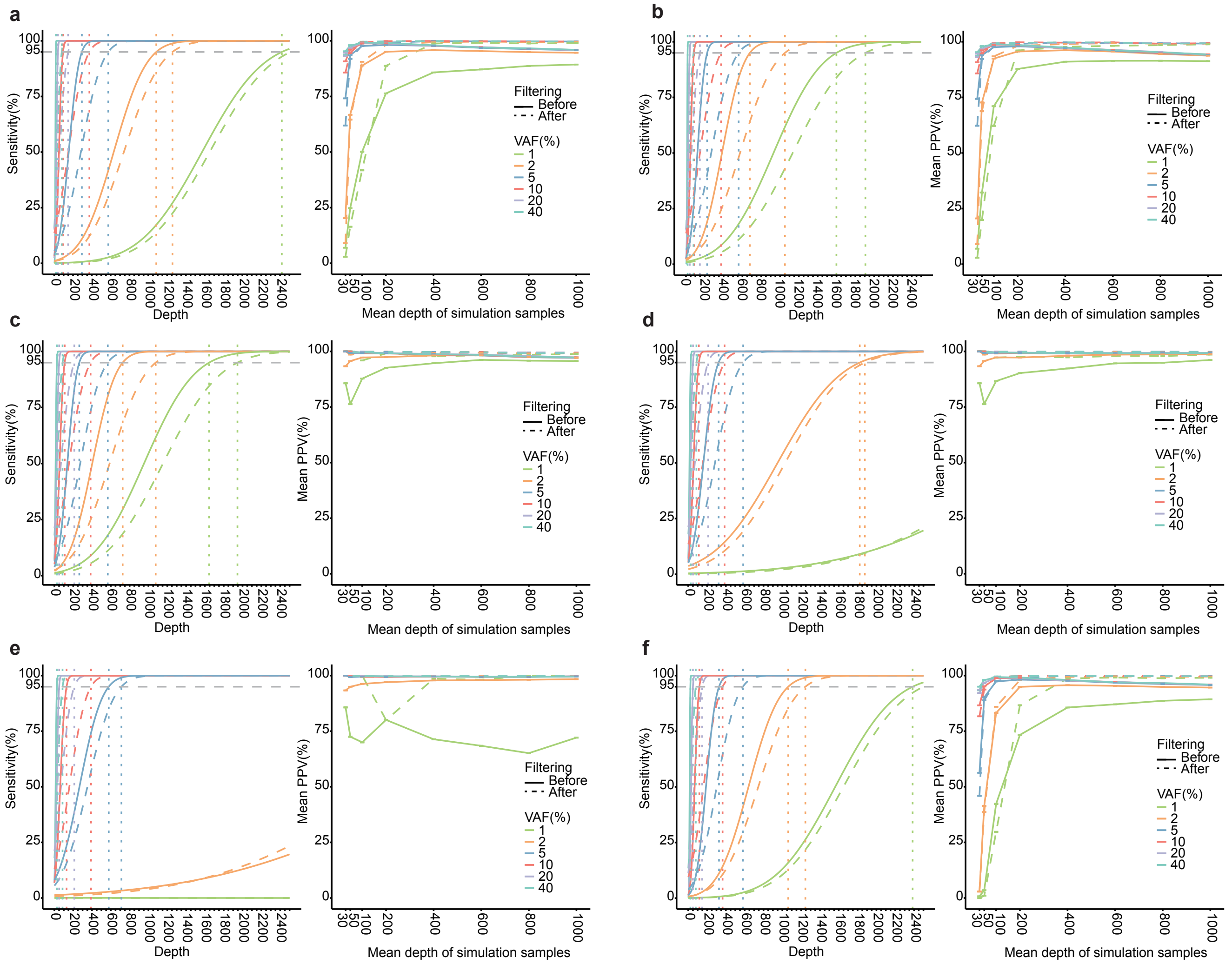

Supplementary Figure 11. LOD and positive predictive value (PPV) for different SNV caller combinations/parameters. (a) Combination of MuTect (High-confidence (HC) mode and default contamination fraction) and LoFreq (default). **(b)** Combination of MuTect (HC mode and contamination fraction (0.00)) and LoFreq (default). **(c)** MuTect (HC mode and contamination fraction 0.00). **(d)** MuTect (HC mode and contamination fraction 0.01). **(e)** MuTect (HC mode and default contamination fraction). **(f)** LoFreq (default). Vertical dotted lines indicate athe read depth to achieve LOD (95% sensitivity). In each case, we also show the results with and without a custom filter, which increases PPV in exchanges for a small increase in depth necessary for a given LOD. Although the option (c) shows excellent performance, it does not achieve perfect sensitivity because some variants are filtered as a result of its stringent filtering criteria. Therefore, we chose to use (a), where LoFreq rescues those filtered variants.

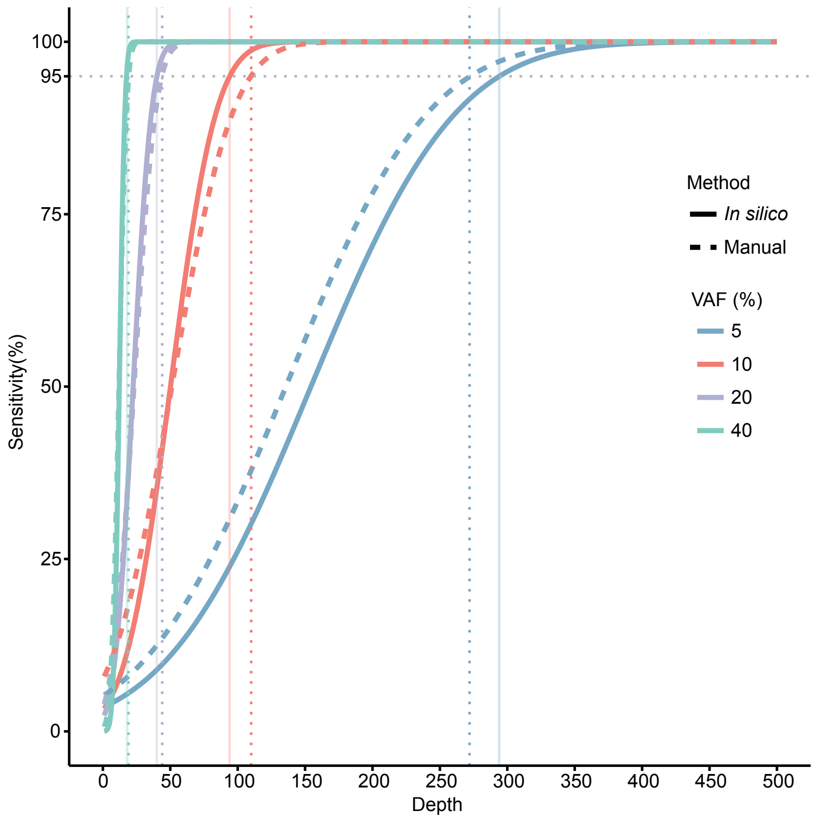

Supplementary Figure 12. Comparison of detection sensitivity between the manual and the *in silico* dilution assays. The lines were interpolated using the probit function. LODs of at allele fractions 40%,20%, 10%, and 5% are 19X, 44X, 110X, and 272X for the manual dilution (experimental mixing of cell lines) and 18X, 40X, 94X, and 294X for the *in silico* dilution, respectively.

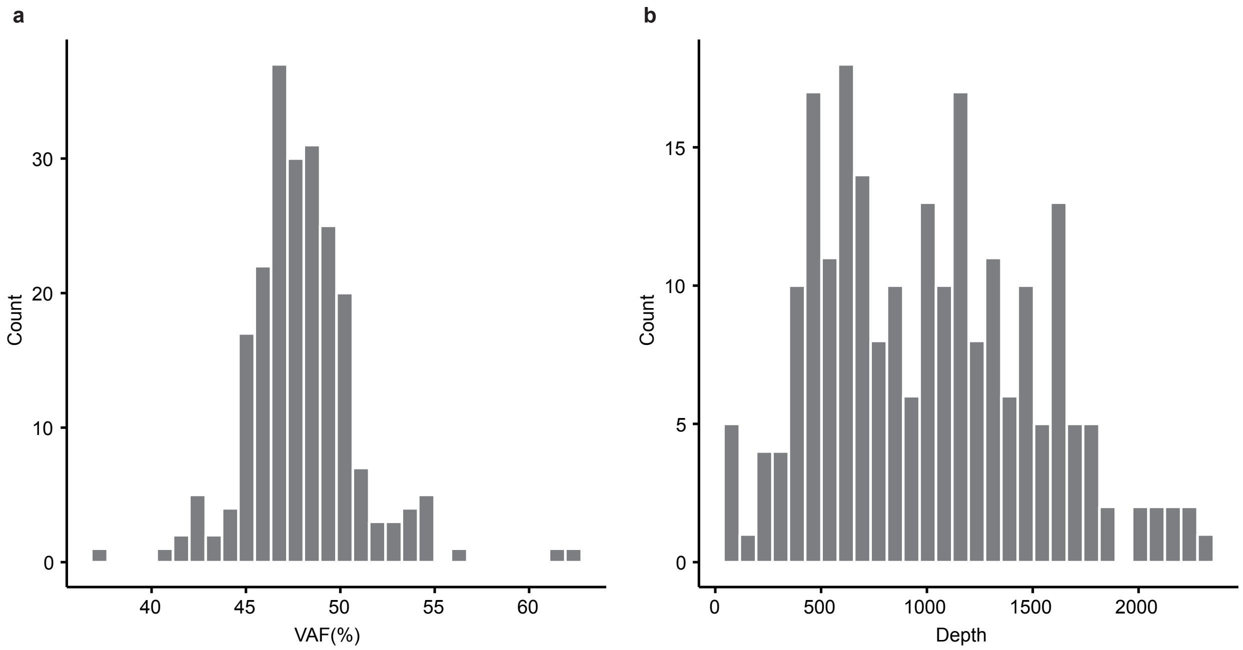

Supplementary Figure 13. Distributions of VAFs and depths at 222 heterozygous SNPs in NA12878. (a) Distribution of VAFs. At these SNPs in the target exonic regions of the panel, the VAFs are centered near 0.5 but there is variability due to variation in depth. **(b)** Distribution of depths. Some SNPs are in hard-to-capture regions and do not many reads.

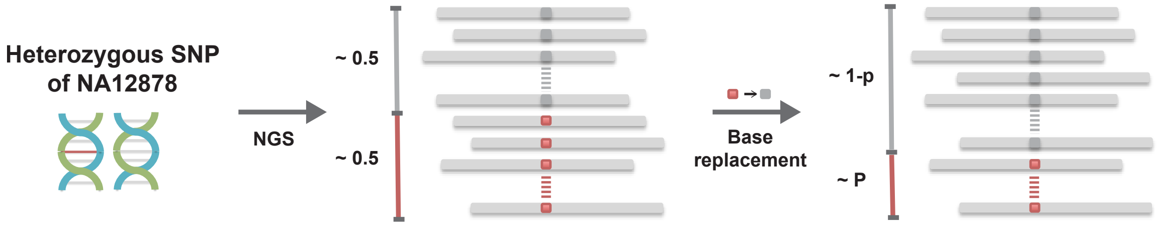

Supplementary Figure 14. Scheme of *in silico* dilution assay. The *in silico* dilution assay is based on the heterozygous SNPs in NA12878. Reads harboring the variant base at the SNP positions are sampled; the variant base is replaced by the reference base with a specified probability to achieve the desired VAF.

Supplementary Table 1. Normal HapMap cell-lines used for manual dilution assay.

Sample name	Dilution assay	Platform
NA07014	Manual	Panel V2
NA10840	Manual	Panel V2
NA18595	Manual	Panel V2
NA18957	Manual	Panel V2
NA18488	Manual	Panel V2
NA18511	Manual	Panel V2
NA18867	Manual	Panel V2
NA18924	Manual	Panel V2
NA19108	Manual	Panel V2
NA19114	Manual	Panel V2

Supplementary Table 2. Classification of variants into three tiers based on the actionable information.

Tier	Definition of tier classification of SNV,Indel,&CNV	Definition of tier classification of fusion
1	Alterations listed as targets of cancer therapeutics in Korean Food Drug Administration (KFDA)/United States Food Drug Administration (USFDA) or reported to be a candidate for clinical trials	Gene-gene fusions of fusion target gene with known partner reported in COSMIC
2	Any alterations reported in COSMIC except tier 1	Gene-gene fusions of fusion target gene with novel partner
3	Any novel alterations, not reported in COSMIC	Gene-gene fusions at non-fusion target region

REFERENCES

1. Bertotti A, *et al.* The genomic landscape of response to EGFR blockade in colorectal cancer. *Nature* **526**, 263-267 (2015).
