# Supplementary material for: Prevalence and detection of low-allele-fraction variants in clinical cancer samples
Source: Nat Commun. 2017 Nov 9;8:1377. doi: 10.1038/s41467-017-01470-y (PMC5680209; doi:10.1038/s41467-017-01470-y)
Supplement: Supplementary file 3 — Description of Additional Supplementary Files [file 41467_2017_1470_MOESM3_ESM.pdf]

Description of Additional Supplementary Files

File Name: Supplementary Data 1
Description: List of target genes.

File Name: Supplementary Data 2
Description: List of samples.

File Name: Supplementary Data 3
Description: Sequencing quality and estimated purity metrics of 5095 samples.

File Name: Supplementary Data 4
Description: List of samples for dPCR validation.

File Name: Supplementary Data 5
Description: List of samples harboring EGFR T790M without EGFR amplification.

File Name: Supplementary Data 6
Description: Clinical information of stageIV colorectal cancer patients.

File Name: Supplementary Data 7
Description: Detection performance of various SNV callers.

File Name: Supplementary Data 8
Description: List of tier 1 alterations.
